# Traditional use and perception of snakes by the Nahuas from Cuetzalan del Progreso, Puebla, Mexico

**DOI:** 10.1186/s13002-016-0134-7

**Published:** 2017-01-21

**Authors:** Romina García-López, Alejandro Villegas, Noé Pacheco-Coronel, Graciela Gómez-Álvarez

**Affiliations:** 1 Laboratorio de Vertebrados, Facultad de Ciencias, Universidad Nacional Autónoma de México, C.P. 04510 Ciudad de México, Mexico; 2 Departamento de Etología, Fauna Silvestre y Animales de Laboratorio, Facultad de Medicina Veterinaria y Zootecnia, Universidad Nacional Autónoma de México, C.P. 04510 Ciudad de México, Mexico

**Keywords:** Ethnozoology, Nahuas, Usage category, Usage value

## Abstract

**Background:**

Indigenous cultures are the result of their adaptation to the natural surroundings, in such a way that, amongst their main features is a set of knowledge, technologies and strategies for the appropriation of nature. In Cuetzalan del Progreso, Puebla, Mexico snakes represent 71.1% of the total local herpetofauna; and in addition to this, different groups of Nahuas have shown to have information of their use of various snake species in many ways. This study was conducted to investigate the traditional uses of snakes in this cultural group.

**Methods:**

Formal and informal interviews were conducted with the inhabitants of the communities. During these interviews, 30 images of the different species of snakes present in the area were presented to the subjects, so that they would recognize them and reveal information about the knowledge they possess on them. A usage analysis was applied to each species considering the following categories: food purposes, medicinal, artisanal and magical-religious. Likewise, the frequency, the diversity and the value of use was estimated for these snakes.

**Results:**

A total of 51 interviews were carried out. The individuals recognized 18 out of 30 images of snakes that were presented. The total of usage categories was five; we found that the magic-religious use was the most mentioned by 32 personas. *Boa imperator* and *Antropoides nummifer* were the species with the highest value of use. More than half of the interviewees mentioned killing snakes because they’re poisonous and aggressive. In the magic-religious aspect the “Danza de los Negritos” is highlighted; this is a local festival, brought by Africans, and alludes to snakes.

**Conclusions:**

This study revealed that snakes are still very important for the culture in Cuetzalan del Progreso, finding that the magical-religious and the medicinal use stand out. On the other hand, the fear and misperception on the toxicity of snakes might represent a potential threat for their conservation. Therefore, it is necessary to carry out a long-term monitoring of the ethno-zoological activities, and develop a sustainable management plan compatible with the cultural characteristics of the natives of the region.

## Background

With time, the state of the world’s ecosystems has deteriorated due to human activities, making it necessary to analyze all the variables that intervene in said deterioration, to recognize which may be modified o eliminated in favor of the environment. Those called “environmental problems” can be described, interpreted and most importantly resolved only through an integrated approach [1]. One of the variables that intervene in the environmental deterioration is that relationship of man with nature. Said relationship of man with animals is affected by the cultural aspects of the different local groups, which are the result of their adaptation to the natural environments, and among the principal characteristics are a great amount of knowledge, technologies and strategies for the appropriation of species. As a result, these aspects press upon the populations of different species that man can utilize in a sustainable way, or endanger their survival [2]. Therefore, it is of a high importance to analyze how the human populations perceive and incorporate those traditional elements to relate to nature [3], and so contribute with effective strategies of conservation [4]. In this sense, those ethnozoological studies that explore the relationships between communities and the utilized fauna as well as a perception they have of the different species are very important.

In Mexico, more than 80% of those areas considered protected are inhabited by indigenous groups, conserving their native language [5]. Within Mexican land most of the worlds’ ecosystems are present, and inside these, we can find countless species of vertebrates that are endemic of the country, with amphibians and reptiles being most important, each with 373 (31%) and 830 (68.9%) species respectively [6]. Wilson and Johnson [7] reported that the highest levels of herpetofauna endemism in Mesoamerica are found in Mexico, with 259 (66.8%) amphibian species and 474 (57.2%) reptile species. Among these reptiles, snakes have a particular importance, and have been considered a sacred deity, associated to the forces of nature due to their unique method of locomotion, similar to the movement of water and lightning [8], by different cultures of the world at different moments [9]. According to De la Garza [9], in Mesoamerica and the western cultures, snakes are closely related to the earth, and symbolize the Great Mother Creator of the Cosmos, which means origin, but also death. The deadly poison in some species of snakes makes them be considered a being of supernatural powers and so, to be worshipped, but also feared. As a consequence, snakes have created a strong aversion and are therefore persecuted by man, being probably that group of animals with the worst reputation [10].

Studies undertaken in rural localities of the northern Mexico, as well as in Brazil, Portugal and Nepal show us that different groups of people have a negative attitude toward snakes, considering them “bad”, and for this reason they should be eliminated; also, there’s the belief that all snakes are poisonous and therefore they should be sacrificed [11–14]. This perception has also been observed in urban centers, including among students [15, 16].

In Mexico, snakes have been traditionally used by different ways, including as food with the boa (*Boa imperator*), snake which the Mexicas called *mazacoatl*, and ate its flesh, considering it softer than any domestic bird [17]. In our days, rattlesnakes are eaten by the native and rural groups of the northwest and central Mexico [18, 19]. Traditional medicine also utilizes these animals, because snakes are considered miraculous animals, in that they heal all kinds of illnesses [20, 21]. Meat, viscera, blood, skin, fangs and rattles are used to cure all types of illnesses, and is also considered a divine and protector animal [22–24]. Especially the *Crotalus* genus is still used to cure skin spots, cancer, ulcers, zits, rashes, facial moles, blackheads, stress, hemorrhoids, heart disease, rheumatism, itching, diabetes and sexual impotence [25, 26]. In other countries, for example Brazil, the flesh, fat and skin of boids and crotalids is consumed for the relief and treatment of rheumatism, arthritis, swellings, and muscular pains in humans [27–29], as well as for domestic animals and against the bite of other poisonous snakes [30, 31]. In northeastern Argentina, the boa is used against chicken pox and measles [32]. In some communities of India, the cobra is used as an animal of veneration and worship, and python flesh for bad vision [33, 34]. In Australia, northwestern groups of natives in Tasmania use the skin and the faeces of poisonous snakes as remedies for bone fractures and back pain [35].

In this same context, in some localities of Mexico, local groups conserve a traditional perception and use of snakes, especially in the State of Puebla, in the municipality of Cuetzalan del Progreso. This place includes 71.1% of the total of the local herpetofauna [36, 37], and 80% of the inhabitants speak the Nahua language [38]. Added to this, the known fact that Nahua groups have the information for the use of different species of snakes [39, 40], we considered it necessary to retake this knowledge also from other localities in the area: San Miguel Zinacapan and Ayotzinapan, in the municipality of Cuetzalan del Progreso, in the State of Puebla, to document the perception and the practices of the usage of snakes in the region.

## Methods

### Study area

**Fig. 1:**
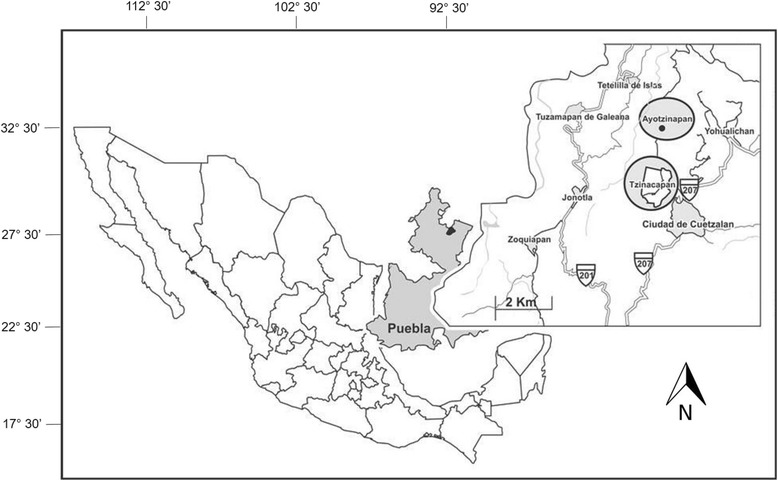
Study area showing the small town of Cuetzalan del Progreso and San Miguel Tzinacapan and Ayotzinapan communities, from which data were collected

Cuetzalan del Progreso is located at Northwestern Puebla (19° 21’ 36″ and 20° 05′ 18″ N, 97° 24′ 36″ and 97° 34′ 05″ W), with altitudes ranging from 320 to 1500 m (Fig. 1) [38]. Three physiographic areas converge here: Sierra Madre Oriental, known locally as the Sierra Norte de Puebla; the Llanura Costera del Golfo Norte, comprising Veracruz, and the Eje Volcánico Transversal. The existence of three distinct physiographic conditions in a 960 km^2^ area gives rise to a variety of landscapes with particular and complex conditions, and to specific geological substrate, soil, weather, vegetation, morphology and geomorphological processes [41]. This area has a warm climate with year-round rain. Cuetzalan is one of the regions with the highest values of precipitation in the country, ranging from 1900 to 4100 mm annually. Due to the irregularity of the topography and the local weather conditions, Cuetzalan has different types of vegetation, such as pine-oak forest, semi-deciduous tropical forest, and cloud forest [36]. San Miguel Tzinacapan has a population of 2 939 inhabitants and Ayotzinapan has one of 1 212; most of these dedicate themselves to corn, coffee and beans agriculture, and to a lower degree to hunting [38].

### Data collection

Samplings were performed from June of 2010 to July of 2011; to obtaining information, the intentional selection of people [42] was used through the Snowball sampling technique [43], “local experts” who had traditional knowledge of local fauna were considered, they were natives of the localities and did field work or hunting. Housewives were also considered with the goal of gathering more detailed information about the food and medicinal uses. In both localities inhabitants were interviewed in a formal and informal manner [44].

The formal interviews were carried out in two ways: the directed and the undirected ones. In the first, they were used to obtain information about a specific topic, the second allowed the informant to lead the course of the interview through a conversation [45]. The interviews consisted in presenting 30 visual stimuli (pictures) of the different species of snakes that are present in the area to the subjects, so that they would recognize them and reveal information about the knowledge they possess on them (like feeding habits, habitat, behavior, reproduction and toxicity) and the use that reptiles receive [46].

### Data analysis

To analyze the data obtained during the interviews, according to Cotton [47], a usage analysis was carried out, considering the following categories: Food use (*F*), for when the snake or any of its parts was eaten; Medicinal (*M*), for when the snake or any of its parts were used as treatment for a disease or affliction that affected the body or soul of the person; Clothing (*C*), for when any part of the snake was used as clothes or as accessories (like earrings made out of vertebrae, leather bracelets, leather belts and others); Artisanal (*A*), for when people make artistic representations of the snakes in any kind of material, or use parts of the snakes as decorative ornaments; Magical-religious (*R*), for when there’s beliefs, myths, superstitions and rites that people perform regarding snakes, in addition to amulet use. With this, the frequency of use, diversity of use and value of use was estimated using the following equations:

The frequency of use was estimated with equation (1):

1 $$ FU=\frac{M{n}_s}{Ni} $$

where *Mn*_*s*_ is the number of mentions per species (*s*) and *Ni* is the number of interviews that were carried out. To estimate the diversity of use for each species, the equation 2 was used:

2 $$ D{U}_s=\frac{C_s}{5} $$

where *C*_*s*_ is the number of categories in which the species (*s*) was mentioned, and *5* is the number of total categories considered in this study. The value of use for each species was estimated with the summation of the value of use for each species in each category, for this the equation (3) was used:

3 $$ V{U}_c=\frac{\sum iM{n}_c}{Ni} $$

where *Mn*_*c*_ is the number of mentions of each interviewed (*i*) for each species in any category of use (*c*), *Ni* is the number of interviews, the subscript is substituted in each one of the categories of use in equation (3).

The risk category in which the species are classified was searched for in the Mexican Official Norm-059 [48], the Red List of the International Union for Conservation of Nature [49], and the protection category in which they are included according to the Convention on International Trade in Endangered Species of Wild Fauna and Flora [50].

## Results

A total of 51 interviews were carried out, 43 of the respondents were men and eight were women, whose ages ranged between 17 and 83 years: 18 young people (Y; 17–39 years), 17 adults (A; 40–59) years and 16 elder (E; 60–83 years). The interviews included 18 agriculturists, six merchants, six housewives, five craftsmen, five teachers, three huntsmen and two dancers; the other six were: three professionals, two students and a painter.

The individuals recognized 18 out of 30 images of snakes that were presented; these snakes were placed in the use categories that were mentioned in the interview. Eleven out of 18 snakes could be found in at least one category of protected species, according to the Mexican laws Nom-059-ECOL-2010, IUCN and CITES (Table 1). With the answers that were given by the respondents it was evident they possessed information on the biology of snakes in aspects of feeding habits (*n* = 38 mentions; Y = 12, A = 14, E = 12), habitat (*n* = 32 mentions; Y = 11; A = 11; E = 10), behavior (*n* = 24 mentions; Y = 9, A = 8, E = 7), reproduction (*n* = 19; Y = 4, A = 8, E = 7) and to a lesser extent on their toxicity (*n* = 11 mentions). Some informants (*n* = 15) indicated that snakes are harmful, because they cause death, wounds, they bite and are poisonous. More than the third part of the interviewees (*n* = 20) pointed out nonpoisonous snakes and referred to them as if they had poison. On the other hand, there were persons (*n* = 5) that considered snakes beneficial, for they end with plagues, give “luck” to crops and eat other snakes.

**Table 1:** Species of snakes used by the inhabitants of the Cuetzalan. The use categories are indicated with the following letters: Food use (F), medicinal (M), clothing (C), artisanal use (A) and magical-religious use (R)

Family/Species	Common name	Use categories	Animal parts utilized	Protection categories		
				Nom-059	IUCN	CITES
Boidae						
*Boa imperator* ^a^	Mazacuate	*F, C, A, R*	Meat, skin	E	-	I, II
Colubridae						
*Conopsis lineata*	Frijolera	*R*	-	-	-	-
*Drymarchon melanurus*	Frijolera	*R*	-	-	LC	-
*Drymobius margaritiferus*	Chirrionera	*M*	Meat	-	-	-
*Geophis* sp.	Coralillo	*M*	Everything	-	-	-
*Imantodes cenchoa*	Nauyaca	*F, M, R*	Fat	SP	-	-
*Leptodeira septentrionalis*	Bejuquillo	*R*	-			
*Adelphicos quadrivirgatum*	Calatera	*R*	-	SP	LC	-
*Coniophanes imperialis*	Chirrionera	*R*	-	-	LC	-
*Lampropeltis triangulum*	Coralillo	*M*	Everything	E	-	-
*Leptophis mexicanus*	Bejuquillo	*R*	-	E	LC	-
*Oxybelis aeneus*	Bejuquillo	*R*	-			
*Pliocercus elapoides*	Coralillo	*M*	Snake	-	LC	-
*Tantilla rubra*	Chirrionera	*R, F, M, C*	Meat, skin	SP	LC	-
*Tropidodipsas sartorii*	Coralillo	*M*	Snake	SP	LC	-
Viperidae						
*Bothrops asper*	Nauyaca	*M, R, C, A, F*	Skin, meat, fangs, vertebrae	-	-	-
*Atropoides nummifer*	Nauyaque	*F, M, R*	Fat	-	-	-
Elapidae						
*Micrurus bernadi*	Coralillo	*M*	Meat and skin	-	-	-

The protection categories are also presented: *E* endangered, *SP* special protection, *LC* low concern, Appendix I lists species that are the most endangered among CITES-listed animals and plants. Appendix II lists species t hat are not necessarily now threatened with extinction but that may become so unless trade is closely controlled.

^a^ *Boa constrictor* as appear in the protection norms due not actualized information

**Fig. 2:**
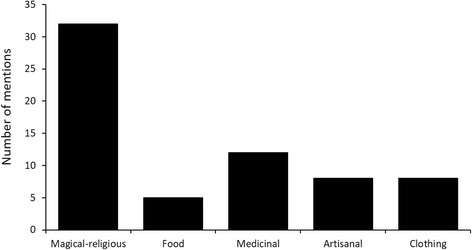
Number of mentions of the informants according to the categories of use of the species registered in Cuetzalan del Progreso, Puebla

**Fig. 3:**
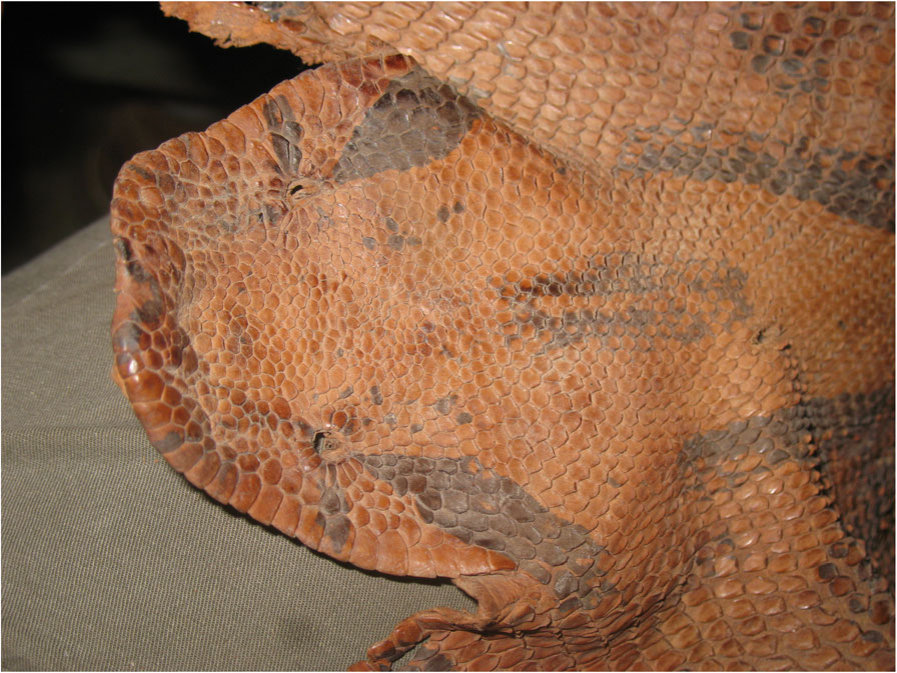
Tanned skin of *Boa imperator* in possession of an inhabitant of San Miguel Tzinacapan, Cuetzalan del Progreso, Puebla

The total number of categories of use was five (Fig. 2). The magical-religious category was the one that was mentioned the most (*n* = 32). *Boa imperator* stands out because of the beliefs that it’s linked to, like for example, that by its mere presence in their land it protects the crops of the inhabitants, also, with *Drymarchon melanurus*, is part of the “Danza de los Negritos”, which is a ritual of African origins and a celebration of the region, that symbolizes the healing of a snakebite with the death of the snake. About *Bothrops asper*, it was mentioned that its fangs are used as good luck amulets and to attract women. *Geophis* sp. and *Tropidodipsas satorii* are conserved inside a corn jar for a week, then they are freed in the countryside and with the, and jar it is believed that protection and good luck will be attracted to their homes. In the medicinal aspect, the informants (*n* = 12) consider snakes useful, for example, they use *Drymobius margaritiferus* to cure all kinds of illness by ingesting its meat, also, the meat of *Atropoides nummifer* is used to cure diabetes and the one of *Botrops asper* to treat rheums. From *Imantodes cenchoa* only its fat is used, it’s smeared in the person’s body to cure cancer, *Tantilla rubra*’s meat is used as a remedy for mosquito bites. A smaller number of informants (*n* = 8) mentioned using snakes as part of their garments and as accessories, they indicated that the skin of *Boa imperator* (Fig. 3) and *Tantilla rubra* are utilized to make belts and shoes, as well as the one of *Bothrops asper* for belts. About the artisanal use, the informants mentioned that that they tend to use *Boa imperator*’s skin to make wallets and knife carriers, *Botrhops asper* is used as an ornament for their houses, previously preparing the skin and their vertebra are used as bracelets. The fans, vertebrae and skin, are prepared by the local tanner, who after perfectly cleaning the material applies chrome and salt to it along 15 days. Finally, the food use obtained the number less of mentions (*n* = 5), pointing mainly to *Boa imperator* and *Bothrops asper*, which is eat smoked and is stewed in chilpozontle and mole (dishes made of chile); *Tantilla rubra* and *Atropoides nummifer* are also eaten, but only as smoked meat.

As far as the evaluation that was made about the use of species, Table 2 shows the values that were obtained, observing that *Boa imperator* and *Artopoides nummifer* have the highest frequency of use, while *Bothrops asper* is the snake that is utilized in the most diverse ways.

**Table 2:** Snake uses in Cuetzalan del Progreso, Puebla

Species	*FU* %	*DU*	*VU*
*Boa imperator*	39.53	0.8	0.55
*Atropoides nummifer*	39.53	0.6	0.51
*Bothrops asper*	18.60	1	0.46
*Tantilla rubra*	6.97	0.8	0.11
*Imantodes cenchoa*	6.97	0.6	0.06
*Conopsis lineata*	2.32	0.2	0.04
*Drymarchon melanurus*	2.32	0.2	0.02
*Drymobius margaritiferus*	2.32	0.2	0.02
*Lampropeltis triangulum*	2.32	0.2	0.02
*Leptophis mexicanus*	2.32	0.2	0.02
*Oxybelis aeneus*	2.32	0.2	0.02
*Adelphicos quadrivirgatum*	2.32	0.2	0.02
*Coniophanes imperialis*	2.32	0.2	0.02
*Geophis* sp.	2.32	0.2	0.02
*Leptodeira septentrionalis*	2.32	0.2	0.02
*Pliocercus elapoides*	2.32	0.2	0.02
*Tropidodipsas sartorii*	2.32	0.2	0.02
*Micrurus bernadi*	2.32	0.2	0.02

*FU* frequency of use, *DU* diversity of use, *VU* value of use

### Inhabitant’s perception of snakes

When inhabitants encounter snakes it usually happens accidentally during their daily activities as, for example, while they are working in the field, when traveling to other towns or when snakes wander into their homes or wander the surroundings, and when they find a snake, they usually kill it. A great part of the interviewees (*n* = 36), mentioned sacrificing snakes because they are harmful, aggressive and poisonous. Most of the villagers mentioned that snakes are killed with a knife, and the animal is taken far away so that the bones do not infect a passer barefoot, or they also tend to put the snake inside a sack once it’s dead, being very careful with its fangs because of the poison. If the snake they encounter is *Boa imperator* or *Bothrops asper*, they use the skin, but if it’s any other species, they dispose of it far away. Snakes are also sacrificed by using a stick stick and beating their head directly, and after they do this they usually hang the snake from a tree or throw it of a cliff so that no one has any risk of harm, as they mention that vertebrae can be dangerous, rotting the skin of the persons who have direct contact with them.

### Danza de los Negritos

**Fig. 4:**
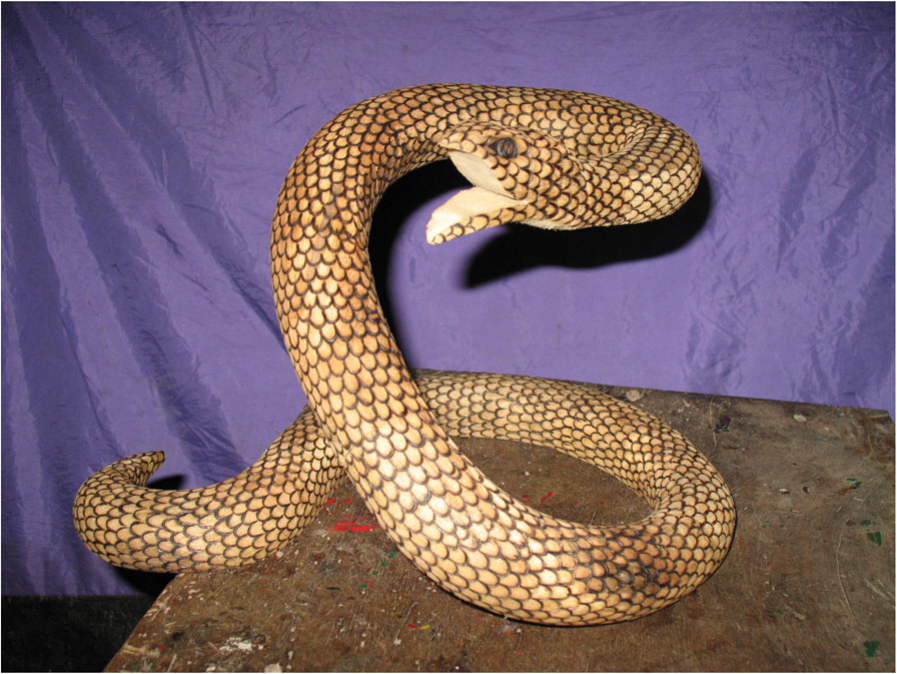
Wood representation of the snake, made by the inhabitants of the area for the “Danza de los Negritos” in Cuetzalan del Progreso, Puebla

In the magical-religious aspect, the “Danza de los Negritos” is part of their cultural traditions. The interviewees mentioned its origins and described it as a dance that was brought by the Africans who arrived to the area as slaves. They believe that the chief of this African culture had been bitten by a snake, and to heal the wound and avoid his death, a ritual was performed and ended with the death of the snake and with the chief being cured. With the passing of time, the ritual, that constitutes an annual festivity, has incorporated various new elements, like a special costume and the representation of the snake made of wood (Fig. 4) or plant roots. The “Danza de los Negritos” is an activity that doesn’t happen during just one day, but in a long process of essays and continued dancing throughout the year, in which the chief of the dance takes charge of the dancers, feeding them during essays and treating them as his own children. The dance representation lasts three days, from the 27th to the 30th of September. This dance is composed by 34 songs plus a ritual that lasts half a day.

## Discussion

Results showed us that the traditional use of snakes is very wide, more than that reported by Martin del Campo [17] used by the natives in pre-Columbian times in the center of Mexico, and by Gutiérrez-Mayén [36] and Blanco-Casco et al. [51] in the study area, indicating a higher use of these animals in the locality. In the State of Morelos, Galeano [52] found that, even with a major pressure of the urban growth, the inhabitants of some communities still preserve wildlife with use value. The same phenomenon happens in the Sierra de Nanchititla, in the State of Mexico, where the method of use of various snakes is reported, particularly of the rattlesnake as food, as ornament, medicine and as pets [19]. This paper reports a very wide use that the community has for snakes, which allows us to propose that the influence of urbanization and interposition of other cultures in the zone is still not a factor that would stimulate its inhabitants to culturally change, still using wild animals. United to this point, it can be observed that the knowledge of snakes the Nahua community has is not limited by the age of the natives, as the young showed us the same knowledge about these animals as the adults.

The magical-religous use of snakes stands out for being the most common one. Gutiérrez-Mayén [36] registered for Cuetzalan, that *Boa imperator* was considered as a beneficial animal, due to de belief that it takes care of the crops and frees them of plagues, as they feed off mice that could destroy their crops. According to the local people *Atropoides nummifer* and *Drymobius margaritiferus* (considered poisonous, without being so) give some kind of protection due to their venom. The medicinal properties of *Boa imperator* mentioned by Sahagún [39] was not documented, probably due to it being more valuable in its magic-religious properties. But the record of nine other snakes, considered medicinal is relevant. Different authors have registered the medicinal importance of snakes in central Mexico, among them. Gómez-Álvarez and Pacheco-Coronel [26], mentioning that snakes of the genus *Bothrops* are used to cure cancer, fatigue, muscular pains and, protectors against evil, information similar to that found in this study; the inhabitants use *Bothrops asper* to cure all types of sickness, including cancer.

The medicinal use of snakes has been registered in all the world to cure a series of diseases similar to those already mentioned. The use of boa in Brazil and Peru [27–29, 53] and in Argentina [32]. In Australia, the cobra (*Naja siamensis*) and the python (*Python regius*) [35]. *Boa imperator* is also used in Brazil to protect and to heal domestic animals and cattle [30, 31]. Some authors have discussed that the curative properties of snakes are related to their mythic and symbolic importance [20, 21], attributes to their association with soil and rain water [9, 54].

The scarce food usage found in this study may be related to the belief that the inhabitants have of some snakes being poisonous, as these interviewed people insisted that they took extreme cautions when consuming these animals. Seri in northern Mexico consume rattlesnakes, but are careful to remove head and tail before, due to the purported toxicity [18]. Sahagún [39] reported the food usage of *Boa imperator* by the Nahua, seen also in this study, together with four other species (*Imantodes cenchoa*, *Tantilla rubra*, *Atropoides nummifer* and *Bothrops asper*). This food usage is also found in South America, where boa is also consumed [53], probably due to the large amount of meat, valued for its high protein content, and its use is frequent among the local natives [55]. For dresses, Blanco-Casco et al. [51] reported the use of the skin of *Boa imperator* and *Bothrops asper* for belts, and the vertebrae for necklaces. We found this same use in Cuetzalan, where the necklaces of vertebrae were the showiest.

The artisanal use is relatively scarce, but those made of carved wood, representing a fer-de-lance (*Bothrops asper*), show us the attention the locals can put in such carvings, with the ventral and dorsal scales perfectly represented.

The artisanal use is relatively scarce, wooden handicrafts made to represent *Bothrops asper*, commonly known as “nauyaca”, show how much attention inhabitants put in this species in particular, it’s evident they possess great knowledge of their external characteristics, with the ability to represent its scales in the ventral and dorsal part perfectly.

The perception the inhabitants have about snakes, notwithstanding that in some cases they consider them useful, is generally negative; killing snakes seems to be an activity arisen from the fear they have of them, though the interviews insisted that snakes are killed for fear of being attacked. Such fear seems to be equal for all species of snakes, poisonous or not; this way of thinking seems to be like that of some inhabitants of northern Mexico [11, 18], from Brazil, [12, 15, 16], Portugal [13], and Nepal [14, 56], where snakes are sacrificed for fear of being bitten, and especially if these animals are large.

In Mexico, the fear of serpents may have arisen a long time ago, probably among the Mexicas who adored the snake (*coatl*), who transmuted them into different gods that managed the world of the living and of the dead [9]. It is probable that myths and beliefs propagate fear and this results in a negative perception of snakes [57, 58]. The perception that snakes are bad and must be eliminated has been observed among students in urban settings [15, 16], which may explain that such disgust or distaste for these animals may have an explanation, not only for the religious-magic symbolism, but also psychologic [59], as the snake represents the origin, but also the destruction and death [9].

Concerning the cosmovision of the “Danza de los Negritos”, it is known that this dance is not only a form of celebration, of happiness, it is also a form of petitioning, as explained by Sten [60]. León-Portilla [61] mentions that the dance is a mystic form of work for their daily life, in this dance time and space change dimension and meaning, once the rites have begun, we leave the daily profane time, to enter the mythic time of the creation, that time that does not flow, which is perennial, and is reached through rituality which communicates us with the gods. The change of time and space is done through the ritual ceremonies, being the dance one of them, and maybe the most important one, the way to talk with the gods, to tell them what we want from them, the form to thank them, to please them, to give them tribute, dance is a prayer. The “Danza de los Negritos” is done in many states of the Mexican Republic, the principal site being the mountain people of the region of the State of Puebla, in the Totonac region of Veracruz and in certain points in Michoacan and Oaxaca. In general terms, it has been considered that the topics that create the majority of the variants of this dance originated during the Colonial Period relate to work in a sugar cane hacienda, and the magic ceremony of killing a snake [62].

## Conclusions

This study shows that snakes are still a very important part of the culture of the inhabitants of Cuetzalan del Progreso, Puebla. The ethnozoological knowledge persists in this area, finding a diversity of uses for this reptile. Snakes are very valuable in the daily lives of its inhabitants because of the magical-religious and medicinal aspects, but at the same time it can expose them to an over-exploitation. Although the food use isn’t that frequent, they can be of great help in the face of an economic crisis, mitigating food shortage; also, they can fight off malnutrition as they provide meat for the inhabitants’ diet.

On the other hand, the fear and misperception of their toxicity, that has a negative consequence in actions the people take, may represent potential threats for their preservation, since a high percentage of them are protected species. Another specific threat is the removal of rare or endangered species. Thus, the factors that are likely responsible for the large scale killing of snakes should beconsidered in the development of biodiversity preservation strategies and in public health. It is necessary to raise awareness and broaden the knowledge about snakes and the healing and prevention of snakebites through educational interventions, as well as the ability to recognize poisonous species. This must be considered in environmental education strategies. Therefore, it is necessary to carry out a long-term monitoring of the ethno -zoological activities of the region, as far as snakes are concerned, to establish baseline studies and develop a short term sustainable management plan that is compatible with the cultural characteristics of the region.
